# Considerations on a new, frameless copper-releasing intrauterine system for intracesarean insertion and its future clinical significance: A review

**DOI:** 10.4274/jtgga.galenos.2020.2020.0003

**Published:** 2020-06-08

**Authors:** Hazal Kutlucan, Recep Onur Karabacak, Dirk Wildemeersch

**Affiliations:** 1 Department of Gynecology and Obstetrics, Gazi University Faculty of Medicine, Ankara, Turkey; 2 Control Research ICC Technologiepark Zwijnaarde, Belgium

**Keywords:** Intrauterine device, intraoperative contraception, preventing expulsions, frameless IUD, insertion technique

## Abstract

Family planning is a system for attaining the desired number of children and enabling a desired spacing between pregnancies. Family planning can be achieved through both the use of contraceptive methods and the treatment of infertility. A woman’s ability to limit her pregnancy has a significant effect on her health.While family planning reduces the rate of unintented pregnancies, it also reduces the number of unsafe abortions. Contraception is an important component of family planning and reproductive health. Among various contraceptive methods, intrauterine devices (IUDs) are very popular because of some of the features of IUDs including being affordable, simplicity of insertion, long duration of action and reversibility. Modern, frameless, copper IUDs contain more copper and their copper content is contained in the solid tubular sleeves rather than in the wire which increases efficacy and lifespan. Immediate postpartum intrauterine device insertion (IPPI) during cesarean section can be considered in women who desire long acting, reversible contraception. Fertility returns instantly after removal of the device and pregnancy rate is not affected. IPPI is a very attractive method, especially for women who have undergone cesarean and require an interval of contraception before getting pregnant again. However, IPPI needs more clinical attention due to many aspects. The advantages remain including the prevention of unintended short interval pregnancies and, by providing an optimal timeframe for post-cesarean uterine recover, can reduce the incidence of the next cesarean delivery. With the publication of international IPPI studies, it will take a place in the range of globally available contraceptive methods, which in this author’s opinion, it deserves.

## Introduction

Family planning provides individuals and couples to get desired number of children and interval between pregnancies. The allowing can be achieved with contraceptive methods (1).

Over three decades ago, an article appeared in the journal Contraception titled: “Immediate postplacental intrauterine devices (IUD) insertion (IPPI): the expulsion problem” (2). The authors had extensive experience with vaginal or cesarean section delivery insertion of several IUDs, having inserted 2646 copper or plain plastic IUDS from June 1, 1974 up to July 1, 1983. IPPI insertion satisfies the basic requirements of any contraceptive method: Ease of insertion, safety of insertion, and high contraceptive effectiveness, but more importantly allows family planning discussions and contraception choices at a time when patient awareness is highest. Postpartum IUD insertion does not enhance the risk of infection or the rate of uterine perforation, has no effect on uterine involution and/or resumption of menstrual activity and does not adversely affect lactation. The authors identified a persisting problem with IPPI, namely poor device retention when compared to conventional interval insertion. Numerous international multicenter clinical trials conducted over the intervening years have repeatedly confirmed the problem. Expulsion rates of up to 70% for the Lippes Loop were reported (2) while a comparative IUD trial conducted by the World Health Organization had to be terminated early precisely because “the predetermined termination indices for expulsions were exceeded at six months”; at one year, the expulsion rates amounted to 41, 44, and 35% for the TCu220C-PP, the Lippes Loop D, and the 7 Cu 200, respectively (3). Finally, a trial conducted by Family Health International in the US reported six-month expulsion rates of 22% and 12% for the Lippes Loop D and the TCu220C, respectively (4).

The American College of Obstetricians and Gynecologists refers to expulsion rates between 10 and 27% (5,6,7,8) but a great variety of expulsion rates are seen in different studies (9), and even higher expulsion rates were found in more recent studies, using copper or levonorgestrel (LNG)-releasing IUDs (10). High expulsion rates may also affect the cost-effectiveness of the method (10).

The clinical and societal benefits of IPPI are clearly evident. However, current methods are viewed by many experts as unacceptable for general use (8). Over the years, numerous attempts have been made to solve the problem of expulsion and IUD displacement primarily focused on establishing a maximal insertion window of 10 minutes post-placenta expulsion. Although these efforts tended to result in more favorable expulsion rates, they remain significantly higher after IPPI than after interval insertion of the same IUD.

In addition to the expulsion problem, there are the high rates of IUD displacement and/or partial expulsion, which result in patient discomfort and early removal of the IUD. Displacement rates are typically not routinely reported in studies or included in expulsion rate determinations. Many IUDs when proper retention and placement is not optimal and, if not completely expelled, will be displaced by uterine contraction and lochia, and can become embedded in the lower uterine segment causing discomfort, cramping and abnormal bleeding. Swati et al. (11) found displacement of the TCu380 IUD in up to 50% of women; many were removed because of abnormal bleeding and pain. For all IUD models, virtually all expulsions are clustered in the first three months after IPPI (2,10).

## The GYN-CS IUD

**Figure 1:**
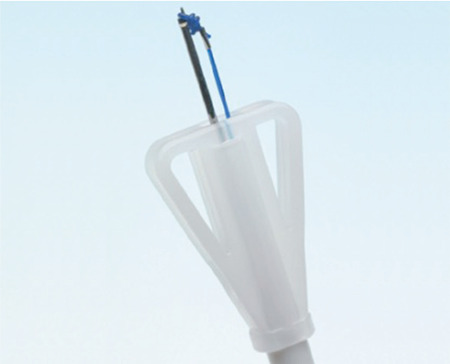
Detail of the tip of the applicator with anchoring knot fixed to the insertion stylet

**Figure 2:**
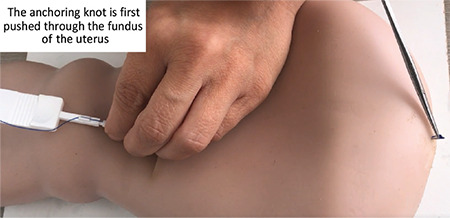
Following puncturing of the fundus, a biodegradable suture is put though the noose of the anchoring knot. Then the anchoring knot is pulled 1 mm below the serosa and fixed to the serosa using the biodegradable suture. (http://www.wildemeersch.com/products/gynefix-cs/video/)

A new surgery-focused approach was devised which eliminates the timing, uterine compatibility issues and clinical complexities associated with IPPI use. The frameless copper-releasing IUD is placed onto the fundal uterine surface via an inserter specifically designed for immediate post-placental delivery after cesarean section (Figure 1). An additional benefit of this new device is that 100% of the copper surface area is available for copper release, the procedure takes advantage of the surgeon’s full view and access to the uterus that is achieved during cesarean delivery and removal of the placenta. The technique consists of the precise placement and fixation of a tiny anchoring knot in the fundus of the uterus immediately following delivery (Figure 2). The entire procedure can be performed in less than four minutes with no discomfort to the patient and minimal surgical risk. Following a series of “proof of concept” studies designed at optimization of the inserter design, the first randomized controlled trial (RCT) was conducted at a tertiary center in gynecology and obstetrics in Turkey. The comparative study, with a follow-up period of three months, compared the frameless, anchored, copper GYN-CS^®^ with the TCu380A IUD in 140 women; 70 in each arm of the study. The approach and insertion procedure of the GYN-CS^®^ is completely novel with respect to IUD placement; the anchor is first pushed through the fundus; then a biodegradable suture is inserted through the noose of the knot after which the knot is withdrawn one millimeter below the serosa and fixed to the serosa using the biodegradable suture. When the suture dissolves after approximately one month, the uterus will have involuted and the knot behaves similarly as after interval insertion. Removal of the GYN-CS^®^, if deemed necessary, has shown to be possible as soon as 30 days post-insertion with no discomfort to the patient or clinical complications. In the study, the TCu380 IUD was inserted using the conventional sponge forceps technique. The RCT results demonstrated the distinct superiority of the anchoring technique as there was only one expulsion (1.4%) in the GYN-CS group while eight total expulsions (11.4%) were observed with TCu380A at the 3-month conclusion of the study (12).

The second study, conducted at the same institution, in 100 women, with follow-up of up to three months, confirmed the validity of the anchoring technique. Only one expulsion was reported (13). The two expulsions, one in each study, were thought to be caused by incorrect physician insertion or by inadvertent or excessive pulling at the tail when setting the anchor or during trimming. Inadvertent traction on the tail could be prevented by trimming the IUD tail in the lower uterine segment at the time of insertion, as opposed to follow-up examination during patient discharge. In the two studies, the single tail of the GYN-CS^®^ could be visualized by subsequent speculum examination in 50-60% of the cases. In the RCT, approximately 25% of the TCu380A IUD strings were visible in the vagina, probably because the single tail of the GYN-CS^®^ is stiffer than the tails of conventional IUDs. If the tail is not visible, the IUD can easily be located in the uterus by abdominal or transvaginal ultrasound examination. The precise positioning of the anchor can be verified in the fundus of the uterus, as the anchor is provided with a tiny stainless-steel marker, highly visible on ultrasound examination.

## Advantages of frameless devices

Frameless devices have an advantage over conventional, framed, t-shape IUDs as they fit uterine cavities irrespective of size or shape. Numerous studies have confirmed that maximal uterine widths are substantially smaller in the majority of women than the width of most conventional IUDs (14). Due to their small size and segmented design, resulting in flexibility and the absence of cross arms, frameless devices have high long-term patient acceptance rates and lack the structure to become embedded. Framed IUDs may be discrepant with the uterine cavity, may displace and embed during involution of the uterus, particularly during prolonged lactation when the uterus becomes extremely small (15).

The most commonly used frameless IUD is active for five years but a frameless device for immediate postpartum insertion, lasting 10 years, has recently been approved and granted CE-marking. A significant advantage of the frameless devices, as they occupy a limited space in the uterus, is that the device can be loaded with a sufficient amount of copper to last up to 25 years. Similarly, a small diameter, high capacity LNG device, loaded with 100 mg of LNG will likely release a sufficient amount of hormone per day to guarantee contraceptive safety for a full 20-year period (16). Both approaches can easily be adapted for use with IPPI procedures. These new, non-hormonal and hormonal, postpartum intrauterine contraceptives will be highly beneficial for women, their clinicals and the general medical community and society in general. They should be welcomed and supported by regulatory authorities for fast-track approval, especially since the long-term efficacy and safety of copper and of LNG is well-established.

The successful implementation of a postpartum program, in addition to solving the expulsion problem, will also depend on taking cost price into consideration. In the USA promotion of the practice of IPPI by the American College of Obstetricians and Gynecologists has been followed by the insurance companies agreeing to reimburse immediate IUD insertion. Currently, 38 states have adopted Medicaid policies to allow the reimbursement of IUDs inserted immediately post-delivery, an extremely welcome initiative. The method will be highly cost-effective if women continue to use the method and thus patient acceptance and comfort are critical factors (10). As the method shows promise, international trials are now planned.

## Implications

In addition to the huge advantages for women, preventing unintended pregnancy, the economic advantages of a highly effective and well-tolerated immediate postpartum method is significant. Therefore, the contraceptive method should be totally reimbursed.

## Conclusion

The development of frameless IUDs has been driven by the growing need to develop high-performing, long-acting, reversible and acceptable contraceptives with a high continuation of use (17). IPPI deserves greater clinical attention as it can provide immediate contraception, prevents repeat unintended pregnancies, and may serve to reduce the incidence or need for secondary cesarean delivery by allowing the uterus to recover optimally post-surgery. Of all the available postpartum birth control methods, IUDs represent the near ideal form, being recommended by physicians and gynecological organizations worldwide. They have the advantage of high effectiveness as well as having an extremely low failure rate, in part because of the lack of concerns of recipient women because these IUDs are well tolerated and are not expelled.

In our department, we have started inserting this useful contraceptive method and will report on the results later.
